# Human Infection with *Rickettsia sibirica mongolitimonae*, Spain, 2007–2011

**DOI:** 10.3201/eid1902.111706

**Published:** 2013-02

**Authors:** José M. Ramos, Isabel Jado, Sergio Padilla, Mar Masiá, Pedro Anda, Félix Gutiérrez

**Affiliations:** Author affiliations: Hospital General Universitario de Elche, Alicante, Spain (J.M. Ramos, S. Padilla, M. Masiá, F. Gutiérrez);; Centro Nacional de Microbiología Instituto de Salud Carlos III, Madrid, Spain (I. Jado, P. Anda);; Universidad Miguel Hernández, Alicante (J.M. Ramos, M. Masiá, F. Gutiérrez).

**Keywords:** Rickettsia sibirica mongolitimonae, rickettsiosis, LAR, spotted fever, rickettsiae, infection, bacteria, Hyalomma asiaticum, ticks, vector-borne infections, lymphangitis-associated rickettsiosis, Spain

## Abstract

Human infection with *Rickettsia sibirica mongolitimonae* was initially reported in 1996, and reports of a total of 18 cases have been published. We describe 6 additional cases that occurred in the Mediterranean coast region of Spain during 2007–2011. Clinicians should consider this infection in patients who have traveled to this area.

The genus *Rickettsia* contains ≈25 validated species of bacteria; another 25 isolates that have not been fully characterized or have not received a species designation have also been described. Signs and symptoms of human rickettsiosis caused by spotted fever group *Rickettsia* spp. include an inoculation eschar (a necrotic area at the site of the tick bite that might not be always present), fever, local adenopathies, and rash, although some variability can be found, depending on the infecting *Rickettsia* species. 

*R. sibirica mongolitimonae* (also spelled *mongolotimonae*) was isolated from a *Hyalomma asiaticum* tick collected in the Alashian region of Inner Mongolia in 1991 (1). Designated *R. mongolitimonae*, the organism was identified as a member of the *R. sibirica* species complex (2), but further phylogenetic analyses grouped it in a cluster separate from other strains of *R. sibirica*. 

The first human case of infection with *R. sibirica mongolitimonae* was reported in France in 1996 (3); since then, 18 additional cases have been described in the literature (4–14). Clinical signs and symptoms of infection are fever; a discrete, maculopapular rash; and enlarged regional lymph nodes, with or without lymphangitis. Although *R. sibirica mongolitimonae* infection causes a mild, not fatal, disease, complications such as acute renal failure and retinal vasculitis have been noted (7,10). We report 6 cases of human *R. sibirica mongolitimonae* infection from the same geographic region of Spain.

## The Study

During July 2007–July 2011, six patients from the Mediterranean coast city of Elche, Spain, who had high fever and inoculation eschars received a diagnosis of infection with *R. sibirica mongolitimonae* (Table). For laboratory confirmation, DNA was extracted from eschars, lymph nodes (fine-needle aspiration), and blood samples by using the QIAamp Tissue Kit (QIAGEN, Hilden, Germany), according to the manufacturer’s instructions. For molecular detection, 200–400 ng of DNA from each sample was subjected to PCR targeting the 23S-5S rRNA intergenic spacer, followed by hybridization with specific probes by reverse line blotting, as described (15). When using the probe for *R. sibirica mongolitimonae*, a positive hybridization signal was obtained from eschar samples from all 6 patients; this result was confirmed by sequencing (100% similarity to a reference *R. sibirica mongolitimonae* strain [GenBank accession no. HQ710799] in all cases in the 357 bp sequenced). To further confirm this result, nested PCR targeting the gene for outer membrane protein A was performed as described (15); these sequences (514 bp) also showed 100% similarity to a reference *R. sibirica mongolitimonae* strain (GenBank accession no. HQ728350).

**Table:** Epidemiologic, clinical, and microbiologic characteristics associated with 6 case-patients infected with *Rickettsia sibirica mongolitimonae*, Spain, 2007–2011*

Characteristic	Patient 1	Patient 2	Patient 3	Patient 4	Patient 5	Patient 6
Patient age, y/sex	67/F	32/M	33/M	42/F	40/F	75/F
Date of illness onset	2007 Jul	2009 Sep	2010 Apr	2011 Mar	2011 Apr	2011 Jul
Type of residence	Rural	Urban	Urban	Rural	Rural	Rural
At-risk activity	Gardening	Working at golf courses	Walking in rural area	Walking in rural area	Excursion by horse	Walking in rural area
Report of tick bite	No	No	No	No	Yes	No
Duration of fever, d	10	4	5	5	6	4
Temperature, °C	38.5	39.5	39.4	39.0	39.0	39.2
Headache	Yes	Yes	Yes	Yes	Yes	Yes
Myalgia	Yes	Yes	Yes	Yes	Yes	Yes
Location of eschar	Scalp	Thigh	Leg	Shoulder	Neck	Leg
Location of enlarged regional lymph nodes	Retroauricular	Inguinal	Inguinal	Supraclavicular	Retroauricular	None
Lymphangitis	No	Yes	Yes	No	No	Yes
Rash	Yes	Yes	No	No	Yes	Yes
Leukocytes, × 10^3^ cells/μL	11.1	2.93	6.40	6.05	NA	NA
Platelets, × 10^3^/μL	540	126	198	217	NA	NA
AST, IU	79	50	48	NA	NA	NA
ALT, IU	65	53	27	NA	NA	NA
C-reactive protein, mg/dL	101	44	46	15.4	NA	NA
Lactate dehydrogenase, IU	642	567	NA	NA	NA	NA
Treatment	Doxycycline	Azithromycin	Doxycycline	Doxycycline	Doxycycline	Doxycycline
Complications	Hyponatremia, lethargy	NA	NA	NA	NA	NA
PCR results						
Eschar	Positive	Positive	Positive	Positive	Positive	Positive
Lymph nodes	NA	Negative	Negative	NA	NA	NA
Whole blood	Negative	NA	NA	NA	NA	NA
IgM/IgG against *R. sibirica mongolitimonae*†						
Acute-phase sample	40/160	<20/<40	<20/<40	NA	40/160	<20/80
Convalescent-phase sample	NA	NA	<20/<40	<20/40	<20/80	<20/80
IgM/IgG against *R. conorii*†						
Acute-phase sample	40/160	<20/<40	<20/<40	NA	40/160	<20/40
Convalescent-phase sample	NA	NA	<20/<40	<20/40	<20/80	<20/40

*NA, not available; AST, aspartate aminotransferase; ALT, alanine aminotransferase. †Determined by using in-house microimmnunofluorescence assay.

Serologic response was analyzed by using an in-house microimmunofluorescence assay for IgG and IgM, performed as described (4); *R. conorii* and *R. sibirica mongolitimonae* were used as antigens, and cutoff values were 1:40 for IgG and 1:20 for IgM. Acute- and convalescent-phase serum samples were obtained from 3 case-patients and single serum samples from the other 3 case-patients. Results for samples from 2 case-patients were negative, but results for the remaining 4 samples showed low to medium titers. Two samples were positive for IgM and 4 positive for IgG (Table). These results are consistent with previous reports (6), in which ≈30% of cases had a positive IgM result and ≈50% had negative or near-cutoff IgG results.

All 6 case-patients lived in Elche and its surroundings (230,112 inhabitants). Three of the cases occurred during the spring, which is when 10/18 cases reported in the literature occurred (4–14). All 6 case-patients had fever (38.5°C–39.5°C), myalgia, and headache; in the cases from the literature, 18/18 patients had fever, 13/18 myalgia, and 11/18 headache. In our study, 1 case-patient was confused and drowsy on arrival at the emergency department. 

All 6 case-patients had a single inoculation eschar develop: 2 on the neck, 2 on a lower limb (Figure), 1 on the scalp, and 1 on an upper limb. Five (83%) case-patients had enlarged lymph nodes in the region from which the eschar drained, as reported for 10/18 (55%) cases from the literature. Three case-patients (50%) had lymphangitis extending from the eschar to the draining lymph nodes (Figure), compared with 6/18 (33%) in cases from the literature. 

**Figure:**
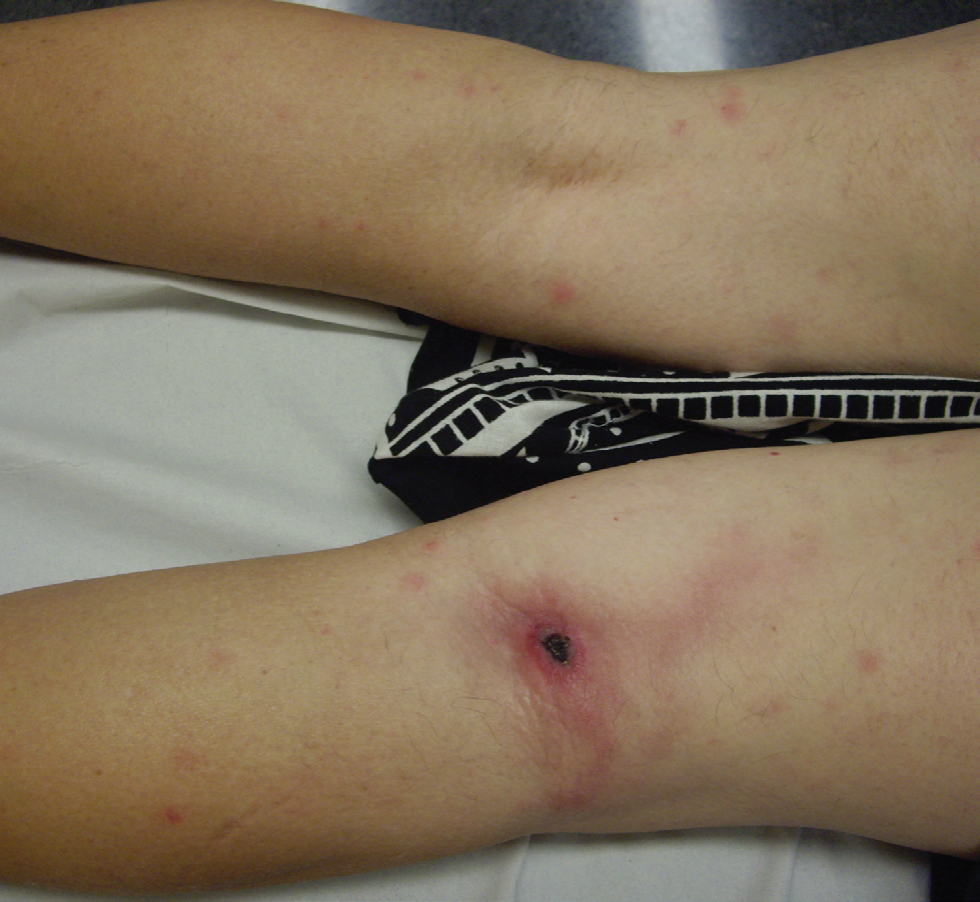
Inoculation eschar on popliteal area and discrete maculopapular elements in patient with lymphangitis infected with *Rickettsia sibirica mongolitimonae*, Spain, 2011.

For 4/6 (67%) case-patients, a generalized maculopapular rash developed on the palms and soles but not the face; for 2 case-patients, a discrete maculopapular rash appeared after 1 day of treatment. These findings are consistent with our review of the literature, which indicated rash occurring in 13/18 (72%) cases. All 6 case-patients recovered without sequelae after antimicrobial drug treatment using doxycycline or azithromycin.

## Conclusions

Fournier et al. (4), who reported 7 cases of *R. sibirica mongolitimonae* infection in 2005, proposed the name lymphangitis-associated rickettsiosis for the disease, on the basis of associated clinical features. However, for the case-patients reported here, the most common clinical signs and symptoms were fever and skin eschar, similar to those from previously reported case series; 5 of the case-patients reported here showed regional lymph node enlargement, 4 rash, and 3 lymphangitis. Because only 24 total cases have been reported and other rickettsioses produce lymphadenopathy and lymphangitis, the term lympangitis-associated rickettsiosis may be unwarranted for this disease.

In our case series, 1 case-patient had mental confusion after 10 days of a febrile disease before hospitalization and was found to be hyponatremic. In this patient, the eschar was located on the scalp, and neither rash nor other clinical clues were suggestive of rickettsiosis. The patient had increased C-reactive protein plasma levels and the highest serologic antibody titers for *R. sibirica mongolitimonae* of the 6 case-patients (Table, patient 1). 

Since *R. sibirica mongolitimonae* was isolated from *H. asiaticum* ticks in 1991 (1), it has been recovered from *H. truncatum* ticks in sub-Saharan Africa (6) and *H. anatolicum excavatum* ticks in Greece (7). These findings suggest a possible association between *R. sibirica mongolitimonae* and *Hyalomma* spp. ticks. However, in Spain, *R. sibirica mongolitimonae* has been detected in 2/8 tick species tested (*Rhipicephalus pusillus* and *Rh. bursa*) at similar percentages (3.7% and 3.6%, respectively) but not from *Hyalomma* spp. ticks (15). Similarly, 1/20 samples of *Rh. pusillus* ticks from Portugal was positive for *R. sibirica mongolitimonae* (8), while testing of other tick species, including *H. lusitanicum, Rh. sanguineus*, and *Rh. bursa,* yielded negative results. That *Hyalomma* spp. (*H. lusitanicum* and *H. marginatum*) ticks have not been found to be infected in Spain does not mean that these ticks are not vectors for *R. sibirica mongolitimonae*. However, data from the literature support the hypothesis that *Rhipicephalus* spp. ticks are a vector for this rickettsia on the Iberian Peninsula, and our findings confirm that this rickettsia species is circulating in Spain, where specific vectors have yet to be described.

In summary, we report 6 cases of human infection with *R. sibirica mongolitimonae* that occurred in the same geographic area of Spain. Our results indicate that PCR of eschar samples is the most useful diagnostic procedure for this pathogen; samples from all 6 case-patients had positive results, while test results for 1 whole blood sample and 2 lymph node samples were negative. However, the limited number of samples does not make it possible to infer specific diagnostic sensitivities.

The epidemiology and pathogenicity of illness caused by *R. sibirica mongolitimonae* infection require further investigation. An active search for the vector of *R. sibirica mongolitimonae* in countries of the Mediterranean region is necessary to complete the epidemiology of this rickettsiosis, which is likely to be more widespread than originally assumed. In particular, clinicians caring for patients who have traveled to the Mediterranean coast of Spain should consider this rickettsiosis in the differential diagnosis.
